# Patient survey in immune thrombocytopenia (ITP): Identifying unmet needs related to treatment and disease control in patients living in the United States

**DOI:** 10.1111/bjh.20257

**Published:** 2025-08-17

**Authors:** Nichola Cooper, Caroline Kruse, Sharon Deneen Morgan, Julie Laurent, Marleni Arvelo‐Saillant, Jean‐Pascal Roussy, Matias Cordoba, Imene Gouia, Lisa‐Anne Schmitt, Erin Reineke, Terry Gernsheimer

**Affiliations:** ^1^ Department of Immunology and Inflammation Imperial College London UK; ^2^ Platelet Disorder Support Association Cleveland Ohio USA; ^3^ Platelet Disorder Support Association Volunteer and ITP Patient Representative Memphis Tennessee USA; ^4^ Carenity Paris France; ^5^ Sanofi, Global Public Affairs Paris France; ^6^ Sanofi, Global Public Affairs New York City New York USA; ^7^ Sanofi, Medical Cambridge Massachusetts USA; ^8^ Sanofi, Health Economics and Value Assessment Gentilly France; ^9^ Sanofi, United States Public Affairs Cambridge Massachusetts USA; ^10^ Sanofi, Scientific Communications Cambridge Massachusetts USA; ^11^ University of Washington and Fred Hutchinson Cancer Center Seattle Washington USA

**Keywords:** bleeding, fatigue, immune thrombocytopenia (ITP), patient survey, quality of life (QOL)

## Abstract

Immune thrombocytopenia (ITP) is a chronic disease with primary therapeutic goals of platelet count recovery to safe levels to minimize active/future bleeding, alongside easing additional symptoms negatively impacting overall patient well‐being with consequent improvement in physical fatigue/energy levels, daily/work‐related activities and social/emotional health. Documentation of this rare disease is important for evaluating real‐world experiences in treatment satisfaction, expectations and unmet needs in disease management. This cross‐sectional, real‐world evidence survey was conducted from 9 February 2023 to 4 April 2023, by the Platelet Disorder Support Association and Sanofi in US adults diagnosed with ITP for ≥1 year. Results showed that although most patients receive and adhere to treatment, lack of sustained efficacy fuels a need for more effective therapy. Patients desired long‐term ITP control and were most concerned with increasing/stabilizing platelet counts and decreasing fatigue and bleeding severity. Better treatment was needed to ease burdensome health‐related quality of life symptoms, especially physical fatigue and anxiety, and decrease effects on daily life and activities. Additionally, involvement in shared decision‐making and engagement in educational, digital healthcare and psychological support were preferred. Overall, ITP patients desired long‐term disease management options that improved platelet counts, minimized bleeding and reduced physical fatigue and anxiety, while also engaging in patient support options.

## Graphical abstract

Key aspects of this survey focused on treatment approach, controlling immune thrombocytopenia (ITP), health‐related quality of life (HRQOL) and patient support. Although most patients were receiving and adhering to ITP treatment, there was a lack of sustained efficacy, fuelling a need for better and more effective treatment options. As anticipated in this chronic disease, patients desired long‐term ITP control and were most concerned with increasing and stabilizing their platelet counts while decreasing fatigue and the risk for severe bleeding. Patients desired better treatment to ease burdensome HRQOL symptoms, particularly related to physical fatigue and anxiety, and to decrease the burden of symptoms on daily life and activities. Additionally, patients wanted to be involved in shared decision‐making along with engaging in educational, digital healthcare and psychological support when offered by their care team.

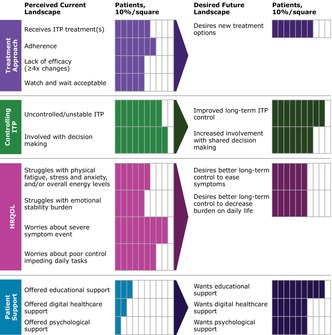

## INTRODUCTION

Immune thrombocytopenia (ITP) is an acquired autoimmune disease characterized by immune‐mediated platelet destruction and impaired platelet production, leading to thrombocytopenia and an increased risk of bleeding events.^1^, ^2^, ^3^ While single episodes followed by an immediate remission have been seen in some patients, ITP is known as a chronic condition (i.e. lasting >12 months) in most adult patients.^1^ The incidence of chronic ITP ranges from 2 to 4 per 100 000 person‐years, with a higher rate in patients over 60 years of age.^4^, ^5^, ^6^ The prevalence of chronic ITP is approximately 10/100 000 people.^6^ Although mainly characterized by thrombocytopenia, which can be severe, the primary symptom is bleeding that can range from mild bruising and mucosal bleeding to severe haemorrhage.^1^, ^7^, ^8^ Patients with chronic ITP have an estimated 5‐year cumulative rate of 1%–2% experiencing intracranial haemorrhage.^7^ Furthermore, patients may be at a higher risk for venous thromboembolism.^1^

Treatment is directed towards increasing the platelet counts to stop active bleeding and reducing the risk of future bleeding.^1^ Corticosteroids are the mainstay of first‐line treatment for ITP; however, adverse effects preclude their long‐term use.^1^, ^9^ Although most patients respond to initial corticosteroid therapy, relapse is common when the dose is reduced, and prolonged exposure leads to significant toxicities.^1^, ^10^ Other first‐line treatments include intravenous immunoglobulins (IVIgs) and anti‐D.^10^ However, not all patients respond to first‐line treatment, and subsequent treatment approaches are frequently necessary. Second‐line treatment options include thrombopoietin receptor agonists (TPO‐RA), fostamatinib and rituximab. While splenectomy achieves durable remissions in 60%–70% of patients, it comes with surgical and post‐operative bleeding risks, and potential long‐term complications such as infections (e.g. sepsis) and arterial as well as venous thromboembolic events.^1^, ^11^

Patients with chronic ITP and/or refractory ITP report significant fatigue, and its severity greatly limits both physical and mental health‐related quality of life (HRQOL).^12^ Symptoms of ITP, such as bleeding and fatigue, as well as the potential side effects associated with treatment, can greatly exacerbate the negative effects on HRQOL. Patients with ITP report more physician visits, take more sick leave from work, lack the ability to perform regular household activities and have worse productivity than non‐ITP controls.^13^ Patient‐reported outcomes have identified and quantified the impact of ITP as a disease and its treatments on daily life and HRQOL; the most commonly reported areas of concern were related to signs and symptoms of ITP, treatment effects, emotional and functional health, work, social and leisure and reproductive health.^14^ In a global, cross‐sectional ITP World Impact Survey (I‐WISh) that researched the impact of ITP on HRQOL, 85% of patients reported reduced energy levels, 77% reduced capacity to exercise and 75% limited ability to perform daily tasks due to their ITP.^15^ Furthermore, most physicians (80%) observed reduced HRQOL for their patients with ITP due to ITP symptoms, 66% citing ITP‐related fatigue as the main cause. In a separate I‐WISh report, patients reported persistent and severe fatigue at a much higher rate than physicians and it was the top symptom patients wanted resolved.^16^ Furthermore, reductions in treatment adherence and limitations in daily functioning were reported due to side effects and bother from ITP treatment.^9^ The treatment burden with corticosteroids is especially high, with >4 times the number of patients reporting highly bothersome side effects compared with other treatments and double the number of patients reducing or stopping treatment consequently.

As ITP is a rare disease, the documentation of disease and treatment management by patients and its impact on HRQOL is important to capture in a real‐world setting. This study examined patient experiences and preferences, treatment satisfaction and expectations and unmet patient needs related to disease management and control based on the outcomes of a survey conducted in adults with ITP.

## METHODS

### Study design, participants and survey questionnaire

A cross‐sectional survey was conducted from 9 February 2023 to 4 April 2023, by the Platelet Disorder Support Association and Sanofi in US adult patients diagnosed with ITP for ≥1 year. Patient recruitment was done via the Platelet Disorder Support Association with email invitations and reminders. Questionnaires were executed by Carenity (Paris, France), which also performed data management and analysis. Only participants who completed the questionnaire entirely were included in the data analyses. During the survey period, participants completed the online questionnaire only once. A data quality check was performed on all completed questionnaires.

The questionnaire was co‐developed by Carenity and Sanofi, with input from a steering committee comprising a professor of immune haematology, a Platelet Disorder Support Association representative and a patient with ITP. The steering committee was responsible for providing input on the study methodology, including feedback on specific survey questions, advising on survey recruitment and assisting in the interpretation of survey results. The questionnaire (Appendix S1) collected information on treatment approach (number of treatment changes, reasons for not following treatment and side effects experienced, treatment expectations and drivers for better treatment), controlling ITP (importance of and factors affecting control, control‐related concerns), HRQOL (aspects of life impacted by poor disease control, impact of watch and wait periods on HRQOL) and patient support (impact of disease management by the healthcare professionals [HCP], involvement in shared decision‐making and patient willingness to be more involved and supportive measures in achieving good disease control).

Survey materials and the study protocol were reviewed and approved by the Western Institutional Review Board‐Copernicus Group (WCG). All patients provided electronic informed consent before participation.

### Statistical approach

Descriptive analyses were performed for each survey question. Qualitative variables were described as the number of respondents and percentages. Quantitative variables were described using mean, standard deviation, 95% confidence interval, median, first and third quartiles (interquartile range [IQR]) and grouping in classes. Open‐ended questions were described as text analyses grouped by themes and number of respondents and occurrences by themes. All statistical analyses were performed using the statistical analysis software R (version 4.3.1).

## RESULTS

### Patients

The survey invitation was sent to 803 Platelet Disorder Support Association adult members with ITP in the United States, which was opened by 561 individuals (69% open rate; Figure S1). Of that portion, 128 clicked on the link (18% click rate) and 103 started the survey. Of the total 103 members who participated in the survey, 23 were excluded due to the following: 20 participants did not complete the survey entirely and 3 were screened out (1 answered ‘No, I am not a US resident living with ITP’ to question 1 and 2 were diagnosed in 2022 [question 9]). Inconsistencies such as too little time spent on survey (<4 min), incoherent dates or answers in open‐ended questions and free fields or the occurrence of several suspicious answers were manually checked, but no participants were excluded from the analysis after a data quality check.

For the 80 patients with ITP who fully completed the survey, 43 (54%) were aged >60 years, 63 (79%) were female and 73 (91%) were White/Caucasian and/or European (Table 1). Median time from first ITP symptoms appearing was 12 years (IQR, 5–20) and median time from first ITP diagnosis was 10 years (IQR, 5–19). Most patients were diagnosed with primary ITP (56 [70%]); secondary ITP was diagnosed in 24 (30%) patients. More patients with secondary ITP were >60 years of age (15/24 [62%]) than with primary ITP (28/56 [50%]). Most patients (77/80 [96%]) were managed by HCPs who were haematologist–oncologists. Nearly half of all 80 respondents (39 [49%]) reported at least two comorbidities and 13 (16%) had no other disease than ITP. Of 80 patients overall, the most frequently reported comorbidity was autoimmune disease in 37 patients (46%). Secondary ITP was mainly caused by an autoimmune disorder in 13 of 24 patients (54%) or immunodeficiency in 3 of 24 patients (13%). In terms of professional employment status, 15 of 80 (19%) respondents were retired. Of the remaining patients, professional employment was not disrupted due to ITP in 57/80 (71%) respondents, but was disrupted by ITP in 8/80 (10%) patients. In 80 total respondents, the most frequently reported ITP‐related symptoms (occurring sometimes or often) were general fatigue (58 [73%]), physical fatigue (54 [68%]) and bruising (50 [62%]; Table 1, Figure S2).

**TABLE 1:** Baseline demographics, patient characteristics and current therapy.

Variable, *n* (%)	ITP patients (*N* = 80)
Age (years)	
18–60	37 (46)
>60	43 (54)
Sex	
Female	63 (79)
Male	17 (21)
Ethnicity	
White/Caucasian and/or European	73 (91)
Black and/or African	3 (4)
Hispanic and/or Latin	2 (3)
Other^a^	4 (5)
Type of ITP	
Primary	56 (70)
Secondary	24 (30)
Managing healthcare professional	
Haematologist–oncologist	77 (96)
General practitioner	7 (9)
Immunologist	3 (4)
Other^b^	3 (4)
Professional employment status	
Retired	15 (19)
Not disrupted by ITP	27 (34) works full‐time
	20 (25) does not work
	10 (13) works part‐time
Disrupted due to ITP	5 (6) works part‐time
	3 (4) does not work
Number of current ITP treatments	
0	31 (39)
1	35 (44)
2	9 (11)
≥3	5 (6)
Current ITP treatment (if receiving treatment)	
TPO‐RA^c^	31 (39)
Corticosteroids^d^	12 (15)
IVIg	6 (8)
Rituximab	6 (8)
Fostamatinib	4 (5)
Splenectomy	3 (4)
Other immunosuppressive drugs^e^	3 (4)
Other^f^	5 (6)
Frequency of ITP symptoms, happened often/happened sometimes = total^g^	
General fatigue	39%/34% = 73% total
Physical fatigue	33%/35% = 68%
Bruising	24%/38% = 62%
Mental health	24%/33% = 57%
Petechiae	10%/33% = 43%
Mental fatigue	8%/34% = 42%
Memory and focus issues	6%/35% = 41%
More severe bleeding	8%/16% = 24%
Other: headache	0%/1% = 1%

*Note*: Patients may have responded to more than one answer per variable.

Abbreviations: ITP, immune thrombocytopenia; IVIg, intravenous immunoglobulin; TPO‐RA, thrombopoietin receptor agonist.

^a^ Other ethnicity included *n* = 1 each Middle Eastern and/or Northern African; Far East or Southeast Asian; Native American, Alaskan Native and/or Indigenous; and prefer not to share/do not know.

^b^ Other healthcare professionals included *n* = 1 each obstetrician/gynaecologist, functional medicine doctor and rheumatologist.

^c^ TPO‐RAs may have included romiplostim, eltrombopag or avatrombopag.

^d^ Corticosteroids may have included prednisone or dexamethasone.

^e^ Other immunosuppressive drugs may have included cyclosporin, azathioprine or mycophenolate mofetil.

^f^ Other ITP therapies were *n* = 1 patient each receiving a new drug through a clinical trial, levonorgestrel‐releasing intrauterine system, hydroxychloroquine, vitamin B12 and required supplemental oxygen.

^g^ Figure showing detailed frequency is reported in Figure S2. Mental health included anxiety, depression, trouble sleeping. Mental fatigue included a decrease in the ability to perform mental processes efficiently. Petechiae was defined as small red or purple spots caused by bleeding into skin. More severe bleeding included heavy menses, nose bleeding and gum bleeds.

### Treatment approach

For 49 patients (61%) currently receiving ITP therapy, the main treatments were TPO‐RA (31 [39%]), corticosteroids (12 [15%]), IVIg (6 [8%]) and rituximab (6 [8%]; Table 1). Fourteen patients (18%) received ≥2 concurrent ITP treatments, including TPO‐RAs and corticosteroids (*n* = 2); TPO‐RA and IVIg (*n* = 2); and combinations of mainly either TPO‐RA and/or corticosteroids with other ITP agents in one patient each. Of 30 evaluable respondents not currently receiving treatment (*n* = 18 primary ITP, *n* = 12 secondary ITP), 24 were not treated based on physician recommendation, two recently finished a cycle of treatment and one respondent each did not want to take proposed treatment, the doctor stated that treatment was not needed, treatment was not affordable or the patient cited thrombocytopenia or splenectomy as reasons. The median time since the last ITP therapy for those not receiving treatment was 3 years (IQR, 0.5–5.8).

Patients changed ITP treatment a median number of three times (IQR, 2–6) during their care pathway. Overall, in 80 respondents, 40 (50%) patients changed their ITP treatment ≥4 times, mainly due to ineffective raises in platelet counts (46 [65%]), previous treatment stopped working (23 [32%]) and the occurrence of symptoms (15 [21%]) and adverse events (15 [21%]). For 20 respondents who indicated ever interrupting or modifying their ITP treatment, their patient profile showed that they had a median duration of ITP of 7.5 years (IQR, 5.0–17.3) and had previously changed treatment a median of four times (IQR, 2–5). The main reasons for not taking treatment in 19 respondents were oversight (11 [58%]), unwanted adverse events (7 [37%]) and/or an unexpected event (5 [26%]). Despite the number of changes reported by patients, 60 patients (75%) declared good adherence to prescribed treatment.

Fifty‐six patients (70%) experienced watch and wait periods (*n* = 25 were >60 years of age), which contributed to deterioration for 31 respondents (55%) and impacted at least one of the following aspects: ITP‐related stress/anxiety (36/56 [64%]), overall HRQOL (30/56 [54%]), physical fatigue (21/56 [38%]) and overall health (14/56 [25%]; Figure S3A). For 36 patients impacted by stress/anxiety, the most worrisome aspects were the risk of experiencing spontaneous severe symptoms (31 [86%]), that daily activities may trigger severe symptoms (27 [75%]) and worsening ITP (23 [64%]; Figure S3B). These worrisome aspects were similar in respondents aged 18–60 and >60 years.

### Controlling ITP

**FIGURE 1:**
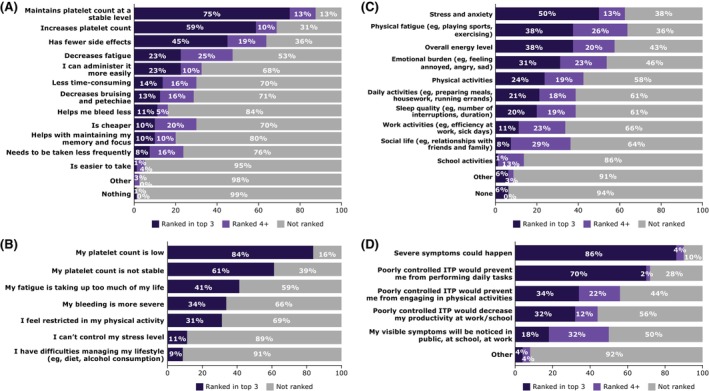
Most important factors for controlling immune thrombocytopenia (ITP) and signifying uncontrolled ITP. (A) What would you expect from a future treatment for ITP? (*N* = 80). (B) Which of the following factors are the most important for you when thinking about controlling your ITP? (*N* = 80). (C) What aspects of your life are most impacted by a poor control of your ITP? (*N* = 80). (D) What aspects of poorly controlled ITP worry you the most? (*n* = 50 indicating an impact on stress and anxiety levels due to poor control of ITP).

Expectations for future ITP treatment were maintaining stable and increasing platelet counts, fewer side effects and decreasing fatigue (Figure 1A,B). Complementing these responses, the most important factors when thinking about controlling ITP included low/unstable platelet counts, fatigue taking up too much of my life and bleeding severity (Figure 1B). Low platelet count remained the most important indicator for controlling ITP irrespective of age group or duration of ITP, and regardless of the presence or absence of symptoms. Patients diagnosed within the prior 1–4 years were more concerned that ITP control was not achievable (Figure S4A). Otherwise, there was no other impact based on disease duration for concerns about maintaining long‐term disease control. Expectations were similar for patients with primary and secondary ITP (Figure S4B).

### Health‐related quality of life

When 80 respondents were asked what it would be like if a patient's ITP was under control, 20 (25%) felt their ITP was under control, whereas 37 (46%) desired less physical fatigue and anxiety related to platelet counts and bleeding. The aspects of life that were most impacted by poor ITP control in 80 respondents were physical fatigue (51 [64%]), stress and anxiety (50 [63%]), overall energy level (46 [58%]) and emotional burden (43 [54%]; Figure 1C).

Of 50 respondents indicating stress/anxiety due to poor ITP control, the most worrisome aspects were the possibility of severe symptoms and prevention of performing/engaging in daily tasks/physical activities (Figure 1D). Reasons for seeking better control of ITP were mainly due to experiencing burdensome ITP‐related symptoms, an inability to perform day‐to‐day activities and hearing about new ITP treatment. In 80 respondents, fears of worsening ITP (39 [49%]) and long‐term side effects (26 [33%]) contributed the most to concerns about long‐term disease control. Patients aged 18–60 years (vs. those age > 60 years) had an elevated concern for the potential of poorly controlled ITP to prevent engagement in physical and daily activities and work/school productivity, along with the visibility of symptoms in public, school or work (Figure S5A). Female respondents were more concerned about the potential restriction of performing daily and physical tasks and that visible symptoms would be noticeable in public, school or work, whereas males were more concerned with the potential for severe symptoms to appear and a reduction in work/school productivity (Figure S5B). Patients with primary ITP (vs. secondary ITP) were more concerned that visible symptoms would be noticeable in public, school or work, whereas patients with secondary ITP (vs. primary ITP) were more concerned that poorly controlled ITP would prevent them from performing daily tasks (Figure S5C).

### Patient support

Globally, in all 80 respondents, satisfaction and actual participation in shared decision‐making were experienced by most respondents; 74 (92%) patients considered themselves involved with their disease management, 70 (88%) felt informed and 66 (83%) trusted their physician. Actual participation in shared decision‐making was reported in 70 (88%) of 80 respondents, and 72 (90%) considered themselves prepared for the discussion. Two‐thirds of patients (54/80 [68%]) want to be more involved in shared decision‐making, irrespective of age. The preference for more involvement was more common in patients with primary ITP (41 of 56 patients [73%]) than in secondary ITP (13 of 24 patients [54%]). Patients desiring more involvement were less satisfied with their ITP treatment.

**FIGURE 2:**
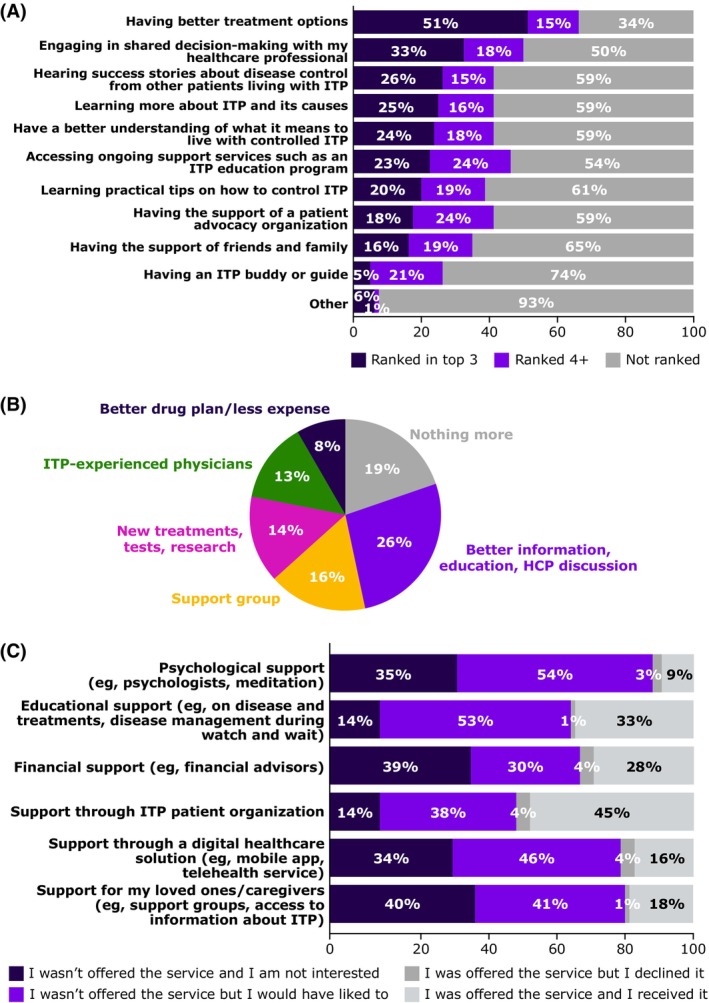
Drivers for better long‐term control and treatment (*N* = 80). (A) What do you think would help you achieve long‐term control of your immune thrombocytopenia (ITP)? (B) What additional help would you have liked to receive during your ITP journey? What are you currently missing? (C) Have you been offered the following services by your healthcare provider(s) since you were diagnosed with ITP?

From 80 respondents, the top drivers for better long‐term control of ITP were cited as having better treatment options (53 [66%]), engagement in shared decision‐making with HCPs (41 [51%]), educational programmes (38 [47%]) and support from patient advocacy organizations (34 [42%]; Figure 2A). Although 19% of patients reported very good medical and support systems with no need for additional help, most patients cited one or more unmet needs (Figure 2B). When support services were offered, patients generally received them (Figure 2C), even though 27 of 80 (34%) overall respondents declared that support services were not offered by their HCP since diagnosis.

## DISCUSSION

**FIGURE 3:**
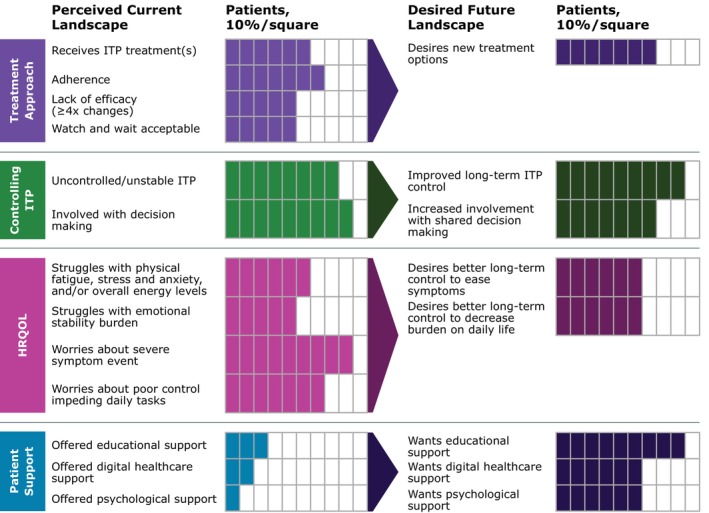
Perceived current and desired future landscapes.

The key aspects of this survey focused on treatment approach, controlling ITP, HRQOL and patient support (summarized in Figure 3). Although most patients were receiving and adhering to ITP treatment, there was a lack of sustained efficacy, fuelling a need for better and more effective treatment options. As anticipated in this chronic disease, patients desired long‐term ITP control and were most concerned with increasing and stabilizing their platelet counts while decreasing fatigue and the risk for severe bleeding. Patients desired better treatment to ease burdensome HRQOL symptoms, particularly related to physical fatigue and anxiety, and to decrease the burden of symptoms on daily life and activities. Additionally, patients wanted to be involved in shared decision‐making along with engaging in educational, digital healthcare and psychological support when offered from their care team.

The presence and impact of fatigue on patients with ITP is increasingly being recognized as an important aspect of the disease. In this study, fatigue was the most reported ITP‐related symptom in the forms of general (73%), physical (68%) and mental fatigue (42%). Secondary to increasing and maintaining platelet counts and decreasing side effects, decreasing fatigue was identified by 48% of patients when addressing what patients would expect from a future treatment for controlling ITP, and physical fatigue was an important contributor to poor ITP control in 64% of patients. While some ITP studies have clearly associated increased fatigue with reduced physical and mental HRQOL,^12^, ^15^, ^16^ its causes have yet to be clarified. While general fatigue was not correlated with platelet count and bleeding scores in an exploratory analysis by Efficace et al.,^12^ it was perceived by physicians in the I‐WISh study as likely to occur in patients with lower platelet counts.^16^ A recent exploratory study by Alesci et al. reported a significant inverse correlation between fatigue and the year of diagnosis (*r* = −0.41; *p* = 0.014), showing that more recently diagnosed individuals with ITP have higher levels of fatigue.^17^ Although neither age nor sex had a relationship with fatigue, there were significant positive correlations between fatigue with depression, obstructive sleep apnoea syndrome and sleep‐related problems (*p* < 0.05). The TPO‐RA Patient experience (TRAPeze) survey results corroborate that fatigue, bruising, petechiae and anxiety about platelet counts are the most common symptoms affecting disease burden in ITP patients across Europe (UK, Ireland, Italy, Netherlands), with fatigue having the greatest negative impact on patient HRQOL.^18^, ^19^, ^20^

In line with other patient‐reported outcomes,^12^, ^15^, ^16^ the most commonly reported challenges with ITP were related to improving control over platelet counts and treatment options that lead to better HRQOL. Patients identified fatigue and reduced energy levels as major symptoms associated with chronic ITP and top symptoms requiring resolution. These effects are likely main contributors for the sequelae of increased physician visits, as well as decreased productivity in work and daily life activities.^13^, ^15^ It is with an optimistic outlook that ongoing and future clinical studies will focus on improving treatment options to increase/maintain platelet counts and decreasing overt bleeding‐related symptoms of ITP, while also considering treatment effects on fatigue and other HRQOL outcomes. Expanded educational, digital healthcare and psychological support from the patient's care team has the potential to benefit patients by engaging patients in learning about their disease to help with identifying and managing the QOL aspects of care.

The study was limited by the inclusion of participants who were recruited via the Platelet Disorder Support Association only, which resulted in selection through a focused patient population that was already actively engaged in learning how to manage their disease with adequate technical skills to respond online to questions. From a demographic observation, most patients were of White/Caucasian and/or European ethnicity and, as generally observed in ITP,^1^ there were more female than male patients. Slightly over half of patients were >60 years of age. Of those included, one‐third of respondents were not currently receiving treatment for ITP (i.e. watch and wait), which may have potentially altered the patient experience in terms of their perception of the disease and satisfaction with treatment and/or physician experience. These results should be interpreted with caution since the results were compiled from an independently developed, non‐validated survey, although they do provide a basis for further evaluation of key aspects of ITP treatment and care.

Overall, patients with ITP continue to seek optimal disease management and long‐term treatment options that improve their platelet counts and minimize treatment‐related side effects (especially bleeding‐related symptoms). In addition, patients are increasingly more active in working to reduce their physical fatigue and anxiety, while better engaging in patient support options when offered.

## AUTHOR CONTRIBUTIONS

N.C., L.A.S., J.L., M.A.‐S., J.‐P.R., M.C., I.G., T.G. contributed to the design of this study. N.C., C.K., S.D.S., M.A.‐S., J.‐P.R., M.C., L.‐A.S., T.G. raised awareness, recruited patients and interpreted data. J.L., M.A.S., M.C., I.G., L.‐A.S., E.R. coordinated data collection and statistical analysis. All authors critically reviewed the draft and approved the final version for publication.

## FUNDING INFORMATION

This study was supported by Sanofi.

## CONFLICT OF INTEREST STATEMENT

N.C. reports grants/research support from Novartis and Rigel; and honouraria/consultation fees from Amgen, Grifols, Novartis and Sobi; and is supported in part by the Imperial College NIHR BRC. C.K. reports uncompensated personal support on behalf of Platelet Disorder Support Association in the form of grants, consulting fees and honoraria from Alpine Immune Sciences, Amgen, argenx, CSL Behring, Novartis, Rigel, Sanofi and Sobi. S.D.M. reports no financial conflicts of interest. J.L. reports current employment with Carenity. M.A.‐S., J.‐P.R., M.C., I.G., L.‐A.S. and E.R. report current employment and current stock shareholder in publicly held company Sanofi. T.G. reports grants/research support from Sanofi; and honouraria/consultation fees from Alpine Immune Science, Amgen, argenx, Bio Products Laboratory, Cellphire, Novartis, Sanofi and Sobi.

## ETHICS STATEMENT

The survey was conducted in full compliance with good clinical practices as defined under the U.S. Food and Drug Administration (FDA) regulations, U.S. Department of Health and Human Services (HHS) regulations and the International Conference on Harmonisation (ICH) guidelines. The protocol and informed consent documents were approved by the Western Institutional Review Board‐Copernicus Group (WCG) on 1 February 2023. All patients provided electronic informed consent prior to participation.

## PATIENT CONSENT STATEMENT

Informed consent was obtained from all participants enrolled in the study.

## CLINICAL TRIAL REGISTRATION

Survey materials and the study protocol were reviewed and approved by the WCG Institutional Review Board.

## Supporting information


Data S1.

## Data Availability

Qualified researchers may request access to patient level data and related study documents including the clinical study report, study protocol with any amendments, blank case report form, statistical analysis plan and dataset specifications. Patient‐level data will be anonymized and study documents will be redacted to protect the privacy of our trial participants. Further details on Sanofi's data sharing criteria, eligible studies and process for requesting access can be found at: https://www.vivli.org/.
