# Supplementary material for: Patient survey in immune thrombocytopenia (ITP): Identifying unmet needs related to treatment and disease control in patients living in the United States
Source: Br J Haematol. 2025 Aug 17;207(3):1038–46. doi: 10.1111/bjh.20257 (PMC12436224; doi:10.1111/bjh.20257)
Supplement: Supplementary file 1 — Data S1. [file BJH-207-1038-s001.docx]

**Patient survey in immune thrombocytopenia (ITP): identifying unmet needs related to treatment and disease control in patients living in the United States**

Nichola Cooper et al.

**SUPPORTING INFORMATION**

*Please see separate PDF document for example of questionnaire*

**FIGURE S1** Flow diagram of immune thrombocytopenia (ITP) patients from the United States (US) included in Platelet Disorder Support Association (PDSA) survey analysis

**FIGURE S2** How often did you experience the following symptoms relating to your ITP over the last 6 months (*N* = 80)?

**FIGURE S3** Impact of watch and wait periods on patient’s day-to-day life.

A. How did watch and wait periods affect your day-to-day life?

B. You indicated an impact of watch and wait periods on your stress and anxiety level. Which aspect(s) of watch and wait periods do you consider the most stressful?

**FIGURE S4** What concerns do you have about maintaining long-term control of your ITP based on time from ITP diagnosis [A] and primary or secondary ITP [B]? N values to the right of each bar represent the total patients providing the ranking for each question; percentages are shown within bars.

**FIGURE S5** What aspects of poorly controlled ITP worry you the most based on age [A] and sex [B], and primary or secondary ITP [C]. (*n* = 50 indicating an impact on stress and anxiety levels due to poor control of ITP)? N values represent the number of respondents indicating an impact on stress and anxiety levels due to poor control of their ITP.
